# A perspectiva emancipatória da literacia em saúde no Brasil: aportes do pensamento freiriano para a translação de saberes em torno de um conceito global

**DOI:** 10.1590/0102-311XPT089824

**Published:** 2025-01-13

**Authors:** Frederico Peres

**Affiliations:** 1 Escola Nacional de Saúde Pública Sergio Arouca, Fundação Oswaldo Cruz, Rio de Janeiro, Brasil.

**Keywords:** Letramento em Saúde, Empoderamento para a Saúde, Autonomia, Educação em Saúde, Health Literacy, Empowerment for Health, Personal Autonomy, Health Education, Alfabetización en Salud, Empoderamiento para la Salud, Autonomía, Educación en Salud

## Abstract

O presente artigo busca desvelar o potencial de aplicação de princípios estruturantes da pedagogia freiriana para uma efetiva translação de saberes e práticas associados ao conceito, globalmente utilizado, de literacia em saúde, em uma perspectiva crítica e emancipadora. Parte-se da constatação de que o processo de apropriação do conceito, no Brasil, se deu de maneira mais utilitarista que crítica, com predominância de iniciativas focadas na tradução e uso de instrumentos para medir literacia em saúde junto a grupos da população, e, dessa forma, acabou por desconsiderar o acúmulo acadêmico, dos atores da prática e dos movimentos sociais nas diferentes regiões do país em torno da aplicação dos princípios e valores associados ao pensamento de Paulo Freire para a promoção emancipatória da saúde de indivíduos e grupos da população. A partir da identificação de princípios básicos do pensamento freiriano, registrados na vasta literatura internacional sobre literacia em saúde, são destacadas possíveis contribuições do pensamento e da pedagogia de Paulo Freire para a consolidação, no Brasil, de saberes e práticas para a promoção da literacia em saúde, em sua perspectiva crítica e emancipatória. E, por fim, sustenta que a literacia em saúde precisa ser considerada como elemento-chave para a promoção da autonomia e do empoderamento, junto a indivíduos e grupos da população, permitindo-os reconhecerem seu direito fundamental à saúde e seu papel enquanto cidadãos, interagindo com outros membros da comunidade, engajando-se em iniciativas para a promoção individual e coletiva da saúde e dialogando com as representações de estruturas sociais circunscritas ao território ou comunidade.

## Abstract (en)

This article seeks to reveal the potential for applying key principles of Freirean pedagogy for the effective translation of knowledge and practices associated with the concept of health literacy followed worldwide, based on a critical and emancipatory perspective. It starts from the observation that the process of adopting the concept in Brazil has been more utilitarian than critical, with initiatives predominantly focused on the translation and use of instruments to assess health literacy among population groups. In doing so, this approach has overlooked the academic contributions, practical actors, and social movements in various regions of the country, regarding the application of principles and values of Paulo Freire’s thought for the emancipatory promotion of health for individuals and population groups. Based on the identification of Freirean thought’s core principles, as reflected in the vast international literature on health literacy, the article highlights possible contributions of Paulo Freire’s thought and pedagogy to the consolidation of knowledge and practices for promoting health literacy in Brazil, in its critical and emancipatory perspective. Finally, it argues that health literacy must be considered a key element for promoting autonomy and empowerment among individuals and population groups, enabling them to recognize their fundamental right to health and their role as citizens. This empowers them to interact with other community members, engage in initiatives for individual and collective health promotion, and engage in dialogue with representations of social structures within their territory or community.

## Abstract (es)

Este artículo busca desvelar el potencial de la aplicación de los principios estructurantes de la pedagogía freiriana para una translación efectiva de saberes y prácticas asociados con el concepto, mundialmente utilizado, de literacia en salud, desde una perspectiva crítica y emancipadora. Se parte de la constatación de que el proceso de apropiación del concepto, en Brasil, se dio de manera más utilitarista que crítica, con predominio de iniciativas centradas en la traducción y en el uso de instrumentos para medir la literacia en salud entre grupos poblacionales. Y que, de esta manera, terminó por desconsiderar la acumulación académica, de los actores de la práctica y de los movimientos sociales, en diferentes regiones de Brasil, en torno a la aplicación de los principios y valores asociados al pensamiento de Paulo Freire para la promoción emancipadora de la salud de las personas y de los grupos de la población. Con base en la identificación de principios básicos del pensamiento freiriano, registrados en la vasta literatura internacional sobre literacia en salud, se destacan posibles contribuciones del pensamiento y de la pedagogía de Paulo Freire para la consolidación, en Brasil, de saberes y prácticas para la promoción de la literacia en salud, desde una perspectiva crítica y emancipadora. Y, finalmente, sostiene que la literacia en salud debe considerarse un elemento clave para promover la autonomía y el empoderamiento, entre individuos y grupos de la población, permitiéndoles reconocer su derecho fundamental a la salud y su papel como ciudadanos, interactuando con otros miembros de la comunidad, participando en iniciativas de promoción, individual y colectiva, de la salud y dialogando con representaciones de estructuras sociales circunscritas al territorio o comunidad.

## Introdução

A literacia em saúde pode ser definida como um conjunto de habilidades e competências que usamos para acessar, compreender, avaliar e dar sentido a informações sobre saúde, com o objetivo de cuidar de nossa própria saúde ou da saúde de terceiros ^1^. São habilidades e competências de que todos os indivíduos dispõem e que aprimoram, ao longo de sua trajetória de vida, a partir de experiências cotidianas, da vida em comunidade, das interações com as instituições sociais e dos processos de aprendizagem, formais e informais ^2^.

Embora o conceito de literacia em saúde, originalmente cunhado em língua inglesa (*health literacy*), tenha sido utilizado pela primeira vez no início dos anos 1970 ^3^, foi somente a partir da segunda metade dos anos 1980, e em particular nos Estados Unidos, no Canadá e na Austrália, que o conceito se consolida como definidor das habilidades e competências utilizadas no processo de significação de informações sobre saúde ^1^ ^,^ ^2^. Inicialmente circunscrito às relações e interações entre médicos e pacientes ^4^, o conceito foi sendo ampliado para dar conta de outros processos interativos relacionados ao cuidado (incluindo o autocuidado) e à prestação de serviços de saúde ^5^ ^,^ ^6^, conformando um modelo mais abrangente, que considera a literacia em saúde em uma perspectiva crítica, fortemente relacionada aos determinantes modificáveis da saúde ^2^ ^,^ ^5^ ^,^ ^7^.

Os estudos sobre diferentes aspectos relacionados à literacia em saúde de indivíduos e grupos da população ao redor do planeta conformaram um campo de saberes e práticas que, embora não se encerre em uma disciplina, apresenta marcos teóricos e conceituais bem definidos ^4^ e que, ao longo do tempo, vêm sendo aprimorados e desenvolvidos, tanto no meio acadêmico quanto junto a diversos atores da prática ^8^ ^,^ ^9^. A crescente literatura sobre o tema, originada nesse processo amplo de construção de saberes e práticas, tem identificado que a literacia em saúde, conforme se desenvolve, propicia, individual e coletivamente, melhores resultados de saúde, o uso mais racional dos serviços e programas de saúde, o aprimoramento das capacidades relacionadas ao autocuidado e melhores resultados de programas de educação e promoção da saúde, entre outros aspectos ^1^ ^,^ ^2^ ^,^ ^5^ ^,^ ^7^ ^,^ ^8^.

No Brasil, o desenvolvimento dos saberes e práticas em torno do conceito de literacia em saúde se deu de forma tardia e peculiar, mesmo quando comparado às experiências de outros países lusófonos, em particular a de Portugal ^6^ ^,^ ^10^. Os primeiros registros de estudos sobre a literacia em saúde, no país, datam da metade dos anos 2000, estando relacionados à compreensão dos processos de significação de informações nos ambientes clínicos, com ênfase na adesão a tratamentos médicos e ao uso de medicamentos, no contexto do autocuidado ^10^. Ao mesmo tempo em que a perspectiva crítica da literacia em saúde - com referências diretas e indiretas à obra e ao pensamento de Paulo Freire - vinha ganhando espaço na literatura acadêmica internacional sobre a temática, os estudos pioneiros no país enfatizavam a tradução, a validação e a aplicação de instrumentos de medida da literacia em saúde (p.ex.: TOFHLA, REALM, s-TOFHLA, NVS etc.) para a avaliação de habilidades e competências de indivíduos e grupos da população brasileira circunscritos a realidades bastante distintas daquelas para as quais esses instrumentos foram pensados ^1^.

Mesmo considerando a importância de tais instrumentos para gerar evidências da necessidade de desenvolvimento da literacia em saúde em determinados contextos ou junto a grupos específicos da população, o enfoque dado pela produção acadêmica nacional aos instrumentos de medida (importados) contribuiu para retardar o processo de construção de um modelo ou abordagem nacional que considerasse todo o acúmulo de conhecimento e desenvolvimento de saberes e práticas associados à perspectiva crítica da literacia em saúde, sobretudo no que diz respeito à influência do pensamento de Paulo Freire e de sua pedagogia emancipadora, pilares reconhecidos por diversos autores de referência como centrais para a promoção da literacia em saúde ^7^ ^,^ ^11^ ^,^ ^12^ ^,^ ^13^ ^,^ ^14^ ^,^ ^15^ ^,^ ^16^.

O presente artigo busca desvelar o potencial de aplicação de princípios estruturantes da pedagogia freiriana para uma efetiva translação de saberes e práticas associados ao conceito globalmente utilizado de literacia em saúde em uma perspectiva crítica e emancipadora.

## A emergência do conceito de literacia em saúde no mundo

Embora não haja um consenso sobre as origens do conceito de literacia em saúde, muitos autores identificam o documento síntese de uma conferência sobre educação em saúde realizada na cidade de Saranac Lake, no Estado de Nova York (Estados Unidos), em 1973 ^3^, como o primeiro documento oficial a utilizar o termo literacia em saúde (*health literacy*), com o intuito de identificar como a educação poderia ser desenvolvida, de forma mais eficaz, para a prevenção de doenças ^9^. Contudo, foi somente a partir da segunda metade da década seguinte, em particular com a publicação do livro *Teaching Patients with Low Literacy Skills* [Ensinando Pacientes com Baixa Capacidade de Alfabetização] ^17^, nos Estados Unidos, que o conceito de literacia em saúde tal qual o conhecemos hoje começou a ser usado para designar o amplo e variado conjunto de habilidades e competências que utilizamos para buscar, compreender, avaliar e dar sentido a informações sobre saúde, visando ao cuidado da saúde, individual ou coletivamente ^1^.

A partir da primeira metade da década de 1990, o conceito de literacia em saúde já era amplamente utilizado e abordado em estudos e iniciativas voltadas para o aprimoramento da comunicação e da educação em saúde em países como os Estados Unidos, o Canadá e a Austrália. Nesse mesmo período e a partir da publicação dos resultados de grandes inquéritos realizados nesses países sobre a alfabetização de adultos, surgem os primeiros instrumentos voltados para uma estimativa rápida da literacia em saúde de indivíduos e grupos da população: o teste de Estimativa Rápida da Literacia em Saúde de Adultos (REALM), em 1993, e o Teste de Literacia Funcional em Saúde de Adultos (TOFHLA), em 1995 ^9^. Duas décadas após os primeiros estudos baseados na aplicação desses testes rápidos de estimativa (métrica) da literacia em saúde de indivíduos e grupos, eles ainda são amplamente utilizados em todo o mundo, com traduções e adaptações para diferentes línguas e contextos ^4^. A maior parte desses estudos se dedica a mensurar a literacia em saúde e associá-la a resultados de saúde, individual e coletivamente ^1^ ^,^ ^9^.

No final da década de 1990, e sobretudo a partir dos resultados da crescente produção acadêmica sobre o tema nos Estados Unidos, no Canadá, na Austrália e no Reino Unido, a Organização Mundial da Saúde (OMS) inclui no seu *Glossário de Promoção da Saúde* ^18^ o verbete literacia em saúde (*health literacy*), cuja definição foi elaborada pelo professor australiano Don Nutbeam, que, dois anos mais tarde, publicou um dos textos seminais do campo de saberes e práticas em torno da literacia em saúde: *Health Literacy as a Public Health Goal: A Challenge for Contemporary Health Education and Communication Strategies into the 21st Century* [A Literacia em Saúde como Meta de Saúde Pública: Um Desafio para as Estratégias Contemporâneas de Educação e Comunicação em Saúde no Século XXI] ^5^. Distanciando-se da maioria dos estudos sobre o tema, circunscritos à métrica da literacia em saúde e fortemente associados a sua dimensão fundamental (linguagem), o texto de Nutbeam propõe um modelo avançado para se desenvolver habilidades e competências para buscar, compreender, avaliar e dar sentido a informações sobre saúde a partir de três níveis distintos: o nível funcional, onde o indivíduo lança mão de habilidades adquiridas ao longo de sua trajetória de vida para significar informações sobre saúde, em benefício do cuidado de sua saúde ou da de terceiros; o nível interativo, em que o indivíduo se vê inserido em uma comunidade em que constrói os vínculos necessários para o cuidado à saúde, assim como para acessar os serviços e programas de saúde disponíveis; e o nível crítico, em que o indivíduo se reconhece como cidadão e membro de um grupo social, capaz de influenciar outros em sua tomada de decisão e de participar ativamente em processos e instâncias da sociedade a fim de promover a saúde, individual ou coletivamente ^5^.

Cinco anos mais tarde, três autores estadunidenses elaboraram um modelo mais avançado e completo para a compreensão e aplicação do conceito de literacia em saúde, a partir de identificação de diferentes domínios em torno dos quais se organizam as diferentes habilidades e competências utilizadas para buscar, compreender, avaliar e dar sentido a informações sobre saúde ^2^. Através desse modelo, é possível organizar as competências e habilidades associadas à literacia em saúde em quatro domínios distintos, facilitando assim a elaboração de estratégias específicas para o seu desenvolvimento: o primeiro domínio é o fundamental, associado à linguagem e às habilidades de leitura, escrita e numeracia (compreender e operar números); o segundo é o domínio científico, associado aos princípios, conceitos, métodos e processos utilizados na produção do conhecimento baseado em ciências, sobretudo as biomédicas; o terceiro é o cívico, relacionado às competências que os indivíduos usam para se reconhecer e atuar como cidadãos em um grupo social, ou junto às instituições que formam a sociedade na qual está inserido; e o quarto domínio é o cultural, associado aos padrões culturais historicamente compartilhados e/ou passados através de gerações, conformando a identidade cultural de um grupo ou sociedade ^2^.

As contribuições de Nutbeam ^5^ e Zarcadoolas et al. ^2^ para o campo de conhecimentos e práticas sobre a literacia em saúde acabaram por consolidar uma perspectiva avançada para os estudos sobre as competências e habilidades usadas no processo de significação de informações sobre saúde. Essa perspectiva colocou em evidência a necessidade de ir além da métrica e dos estudos de ranqueamento da literacia em saúde baseados principalmente na aplicação dos instrumentos de estimativa rápida como o REALM e o TOFHLA, para considerar as estratégias necessárias para o desenvolvimento de habilidades e competências voltadas à promoção da autonomia e do empoderamento de indivíduos e grupos da população, visando ao cuidado de sua saúde ou da de terceiros ^4^ ^,^ ^7^ ^,^ ^13^, assim como facilitando seu papel como agente ativo nos processos individuais e coletivos voltados para a promoção da saúde ^1^ ^,^ ^8^ ^,^ ^12^.

Em diversos países, a crescente e vasta produção acadêmica sobre a literacia em saúde contribuiu para a formulação e implementação de uma série de programas e políticas públicas para a promoção da literacia em saúde junto a indivíduos e grupos da população. Nos Estados Unidos, subsidiaram o *National Action Plan to Improve Health Literacy* [Plano de Ação Nacional para Melhorar a Literacia em Saúde], de 2010; na Austrália, a *National Preventive Health Strategy 2021-2030* [Estratégia Nacional de Saúde Preventiva 2021-2030], de 2021. Em países como Áustria, Irlanda, Itália, Espanha e Reino Unido, contribuíram para a criação de políticas e programas nacionais de literacia em saúde. Em Portugal, possibilitaram a inclusão do conceito de literacia em saúde no *Plano Nacional de Saúde 2012-2016* (de 2012), a criação de um Programa Nacional de Educação para a Saúde, Literacia e Autocuidados (Despacho nº 3618-A, de 2016) e, posteriormente, a elaboração do *Plano de Ação para a Literacia em Saúde 2019-2021* (de 2018).

Já em relação ao Brasil, embora não exista uma política ou programa nacional para a promoção da literacia em saúde, há princípios associados ao conceito presentes na Política Nacional de Promoção da Saúde (PNPS, de 2006, atualizada em 2017), na Política Nacional de Alimentação e Nutrição (PNAN, de 1999, atualizada em 2011) e na Política Nacional de Atenção Básica (PNAB, de 2006, atualizada em 2017) ^1^.

No texto da PNPS, as diretrizes voltadas ao desenvolvimento de habilidades e competências individuais e coletivas para a promoção de empoderamento e fortalecimento de ações comunitárias incluem a necessidade de um esforço educacional integrado, orientado pela literacia em saúde dos indivíduos, visando a melhoria das condições de saúde e a diminuição das iniquidades sociais em saúde ^1^, circunscrevendo tais diretrizes aos domínios cívico e cultural ^2^ e ao nível interativo/comunicativo da literacia em saúde ^5^.

Com relação à PNAN, entre as diretrizes voltadas para a promoção de hábitos alimentares saudáveis, incluem-se o desenvolvimento de habilidades e competências individuais e coletivas para o autocuidado e o exercício da autonomia, dois princípios básicos circunscritos ao conceito de literacia em saúde, em sua perspectiva ampliada ^2^ ^,^ ^5^. Também se destaca a necessidade de trabalhar com práticas problematizadoras e construtivistas, referenciadas na realidade local, que considerem os contrastes e as desigualdades sociais que interferem no direito universal à alimentação, alinhando essa diretriz ao domínio cívico da literacia em saúde ^2^. Ainda no texto da PNAN, destaca-se a necessidade de fortalecimento (ou ampliação) da autonomia dos indivíduos para realizarem escolhas alimentares informadas, a partir do conhecimento dos problemas que os afetam, aproximando essa diretriz do conceito de literacia crítica em saúde (ou nível crítico da literacia em saúde) ^5^.

Por sua vez, a PNAB apresenta, em seu conjunto de diretrizes, o desenvolvimento de habilidades e competências para a promoção da autonomia, individual e coletivamente, permitindo aos indivíduos uma participação ativa e qualificada em fóruns e espaços decisórios próprios da atenção primária à saúde, como, por exemplo, os conselhos (distritais, municipais ou estaduais) de saúde. Peres et al. ^1^ trazem implicitamente o peso dado ao desenvolvimento dos domínios cívico e cultural da literacia em saúde como estratégia para a promoção da participação social.

Já no que se refere à tradução do conceito de *health literacy* para a língua portuguesa, é possível identificar como marco de referência o *Estudo Nacional de Literacia* ^19^ em Portugal, em 1995, que reproduziu, naquele país, iniciativa baseada nos *Inquéritos Nacionais de Alfabetização de Adultos*, realizados anos antes nos Estados Unidos, no Canadá e em alguns países europeus, como a Alemanha, Suécia e Polônia ^20^. A partir do final da década de 1990 e, sobretudo, do início dos anos 2000, o conceito passou a ser mais frequentemente registrado em estudos sobre a literacia em saúde de indivíduos e grupos da população portuguesa, principalmente aqueles relacionados à dimensão fundamental e aos contextos clínicos e ambulatoriais ^6^ ^,^ ^21^.

## A emergência do conceito de literacia em saúde no Brasil

Já no Brasil, os primeiros estudos sobre a literacia em saúde de indivíduos e grupos da população datam de meados da década de 2000, inicialmente utilizando os termos alfabetização e letramento em saúde como traduções do conceito de *health literacy*, e igualmente circunscritos à dimensão fundamental e aos contextos clínicos e ambulatoriais. Segundo Peres ^10^, os estudos sobre literacia em saúde publicados na literatura acadêmica nacional até a segunda metade da década de 2010 utilizaram, prioritariamente, os conceitos de alfabetização ou letramento em saúde, e registraram, em sua maioria, resultados da aplicação, adaptação e validação de instrumentos de medida da literacia fundamental (como os acima identificados), assim como estudos de legibilidade de materiais escritos e revisões (narrativas, predominantemente) da literatura internacional.

O conceito de alfabetização em saúde, tal como registrado na literatura acadêmica nacional, vincula-se fortemente aos estudos sobre a influência dos anos de escolaridade sobre a capacidade dos indivíduos de compreender informações sobre saúde e de tomar decisões baseadas nessa compreensão. Nesses estudos, o grau de alfabetização em saúde era, predominantemente, associado à capacidade de interpretação de textos escritos (legibilidade) ou orientações médicas, à capacidade de adoção de dietas e/ou práticas alimentares saudáveis e ao cuidado de condições crônicas de saúde ^10^.

Já o conceito de letramento em saúde surge, na literatura de referência nacional, com uma alternativa mais abrangente à alfabetização em saúde, circunscrevendo habilidades cognitivas e sociais que permitem aos indivíduos acessar, compreender e utilizar as informações disponíveis como estratégias para promover e manter uma boa saúde ^10^. Mas, apesar de ampliar o escopo de análise para além das habilidades de leitura e escrita, os estudos sobre o letramento em saúde registrados na literatura acadêmica nacional ainda estão muito vinculados ao domínio fundamental da literacia em saúde, incorporando de maneira pontual os fundamentos e princípios que, há mais de duas décadas, conformam a perspectiva crítica/avançada do conhecimento internacional sobre literacia em saúde ^1^ ^,^ ^2^ ^,^ ^5^ ^,^ ^9^ ^,^ ^10^.

Somente a partir do final da década de 2010 (e com mais ênfase a partir do início da pandemia de COVID-19) é que se observa, na literatura acadêmica nacional, a crescente aplicação do conceito de literacia em saúde a partir de uma perspectiva mais ampliada e avançada, embora a maior parte desses estudos ainda se relacione à métrica e ao ranqueamento, a partir da aplicação de instrumentos de estimativa rápida (como o REALM e o TOFHLA) e de inquéritos internacionalmente validados (como o HLS-EU e o HLQ), traduzidos para o português do Brasil ^10^.

A perspectiva mais abrangente e crítica, frequentemente associada ao conceito de literacia em saúde, e tal qual registrada na (ainda) incipiente literatura de referência nacional, contempla análises para além das capacidades individuais e cognitivas dos indivíduos, reposicionando o olhar sobre espaços de práticas sociais e culturais, constituídos a partir da interação dos indivíduos com seu grupo social e com as instituições circunscritas, incluindo sistemas de saúde ^7^ ^,^ ^10^ ^,^ ^22^. Nessa perspectiva, a literacia em saúde adquire um papel fundamental para a compreensão do cuidado e da promoção da saúde, sobretudo considerando as novas e crescentes exigências de compreensão e uso de informações sobre saúde na vida cotidiana dos indivíduos e grupos da população, influenciando o acesso e utilização de serviços, programas e sistemas de saúde e, por conseguinte, se posicionando como um elemento estratégico para a promoção de autonomia nos processos de tomada de decisão informada sobre saúde ^1^ ^,^ ^2^ ^,^ ^5^ ^,^ ^14^ ^,^ ^22^ ^,^ ^23^.

### Lacunas identificadas no processo de adaptação e aplicação do conceito de literacia em saúde no Brasil

Considerando os antecedentes anteriormente expostos e partindo do pressuposto - consubstanciado por recente revisão da literatura de referência nacional ^10^ - de que os estudos sobre a literacia em saúde no Brasil ainda articulam sua perspectiva crítica de maneira marginal e episódica, é necessário olhar para o processo de adaptação e aplicação do conceito de *health literacy*, no país. Primeiro, identificando lacunas deixadas nesse processo de tradução e uso do conceito para explicar fenômenos relacionados à significação de informações sobre saúde em diferentes contextos. E, em seguida, apontando para caminhos possíveis a serem percorridos para a promoção da literacia em saúde, em sua perspectiva mais crítica e emancipatória, possibilitando aos indivíduos e grupos da população brasileira tomar decisões informadas e participar, de forma ativa, dos processos relacionados ao cuidado e à promoção de saúde, individual e coletivamente.

Curiosamente, no Brasil, pátria de Paulo Freire - referência globalmente reconhecida e vinculada a processos de desenvolvimento de habilidades e competências emancipatórias -, o processo de apropriação do conceito de literacia em saúde se deu de maneira mais utilitarista do que crítica, com a ampla predominância de iniciativas focadas na tradução - sem nenhuma ou pouca preocupação com a translação de saberes - de instrumentos de métrica da literacia em saúde validados e em sua aplicação junto a grupos da população brasileira, em particular usuários de serviços e programas de saúde ^1^ ^,^ ^10^. Importante destacar, nesse ponto, que consideramos, para fins de sustentação das ideias apresentadas neste manuscrito, a translação de saberes como uma estratégia de tradução de conhecimentos, devidamente contextualizados, a partir das normas e condutas culturais dos grupos que os originaram - ou seja, a partir da consideração do caráter indissociável entre texto e contexto ^1^ ^,^ ^2^.

Considerando o exposto, instrumentos elaborados em contextos sociais e culturais bastante diferentes em relação àqueles identificados no conjunto da população brasileira, diversos e heterogêneos por natureza, não devem servir como parâmetro de métrica para qualificar a literacia em saúde de indivíduos e grupos da população. No máximo, podem se habilitar como ferramentas pontuais, e de escopo limitado, para identificar possíveis tendências e a necessidade de desenvolvimento de habilidades e competências, individual e/ou coletivamente, desde que devidamente adaptados e transladados à realidade brasileira. Porém, uma recente revisão da literatura nacional de referência sobre literacia em saúde mostra que a aplicação no Brasil de tais instrumentos validados internacionalmente se dá a partir da simples tradução de conteúdos e escalas para o português brasileiro, com poucas experiências incluindo etapas de validação prévia ou pré-testes junto a indivíduos e grupos da população ^1^ ^,^ ^10^.

Ao invés de priorizar a tradução e a validação de instrumentos de métrica da literacia em saúde elaborados em contextos diversos do nacional, os esforços para a promoção da literacia em saúde no Brasil poderiam se apropriar do imenso acúmulo acadêmico, dos atores da prática e movimentos sociais nas diferentes regiões do país em torno da aplicação dos princípios e valores associados ao pensamento de Paulo Freire para a promoção emancipatória da saúde de indivíduos e grupos da população brasileira. Uma rica articulação de saberes e práticas que, entre outras importantes contribuições, acabou por conformar, no país, o campo da Educação Popular em Saúde ^24^, que, embora não seja objeto do presente artigo, compartilha princípios e valores muitos próximos daqueles que orientam a perspectiva crítica/avançada da literacia em saúde ^25^.

É importante destacar que muitos autores ao redor do planeta reconhecem a contribuição de Paulo Freire, em especial de *Pedagogia do Oprimido* ^26^, no processo de construção da perspectiva crítica/ampliada da literacia em saúde, sobretudo no que tange à centralidade da redução de assimetrias no processo de significação comum de saberes muitas vezes distintos - tal qual distanciados se encontram o conhecimento biomédico e a realidade daqueles que mais o necessitam, por exemplo ^2^ ^,^ ^7^ ^,^ ^12^ ^,^ ^14^. Sua obra é citada há, pelo menos, quatro décadas, em centenas de artigos sobre educação e alfabetização, principalmente devido ao sucesso pedagógico de seu método na promoção da literacia (em sua concepção mais ampla, e não restrita apenas à saúde) junto a comunidades tradicionalmente desfavorecidas ^14^ ^,^ ^27^ ^,^ ^28^.

No campo de saberes e práticas criado em torno do conceito de literacia em saúde, a obra de Paulo Freire se estabelece como uma referência importante e amplamente considerada, principalmente pelo pressuposto largamente aceito de que qualquer intervenção para a promoção da literacia em saúde, com legítima colaboração entre os membros da comunidade e profissionais de saúde, deve partir de um encontro convergente de visões de mundo e experiências vividas por ambos os grupos, ou seja, daquilo que está delimitado no conceito freiriano de espaços de significação comum ^1^ ^,^ ^14^ ^,^ ^27^ ^,^ ^28^ ^,^ ^29^. Tal abordagem proporciona um maior envolvimento da comunidade nas decisões individuais e coletivas sobre saúde, ao mesmo tempo que permite aos profissionais de saúde assumir um papel mais ativo na comunidade que servem, gerando um sentido de legítima colaboração cidadã, que, por sua vez, acaba contribuindo para a melhoria da saúde da comunidade - uma lógica que se convencionou classificar como perspectiva avançada, ou crítica, da literacia em saúde ^1^ ^,^ ^2^ ^,^ ^5^ ^,^ ^14^ ^,^ ^28^.

## Aportes de inspiração freiriana para a translação de saberes em torno de um conceito global, no Brasil

As contribuições do pensamento e da pedagogia de Paulo Freire para a consolidação, em diferentes partes do planeta, de uma perspectiva crítica da literacia em saúde são evidenciadas, de maneira direta e indireta, na vasta literatura internacional de referência sobre o tema ^7^ ^,^ ^11^ ^,^ ^12^ ^,^ ^13^ ^,^ ^14^ ^,^ ^15^ ^,^ ^16^. Desde a ruptura com os modelos transmissionistas de desenvolvimento de habilidades e competências, que muito se aproximam do que Paulo Freire denominava por “educação bancária” ^26^, até o cuidado com a adequação sociocultural de estratégias para a promoção da literacia em saúde, imediatamente associado ao que o educador brasileiro chamava de “leitura do mundo” ^30^ ^,^ ^31^, são diversas as referências de inspiração freiriana na sustentação dos princípios estruturantes da perspectiva crítica da literacia em saúde ^2^ ^,^ ^5^ ^,^ ^7^.

Ao trazer elementos do pensamento freiriano para sustentar a importância da perspectiva crítica da literacia em saúde, Matthews ^12^ lembra que o simples acesso a informações sobre saúde, por mais compreensíveis que sejam, não é suficiente para promover mudanças de comportamento. Para a autora, tais mudanças exigem uma abordagem mais crítica da literacia em saúde que permita aos indivíduos tomarem consciência das desigualdades historicamente produzidas e reproduzidas por estruturas antiéticas de poder e de opressão, reconhecendo que sua condição de saúde é resultante de um processo de determinação social, econômica, educacional, cultural e ambiental ^12^. Dessa forma, ela aproxima a promoção da literacia em saúde (em sua perspectiva crítica) da pedagogia emancipatória de Paulo Freire, na qual “*a leitura do mundo precede a leitura da palavra*” ^30^ (p. 9), ao mesmo tempo em que a distancia das estratégias de métrica e ranqueamento da literacia em saúde, a partir de instrumentos internacionalmente validados, fortemente vinculados ao domínio fundamental ^1^ ^,^ ^12^.

Como contribuição para se pensar a literacia em saúde no Brasil a partir de uma perspectiva crítica e emancipatória, identifica-se, a seguir, alguns pontos de convergência entre o pensamento de Paulo Freire e os saberes e práticas necessários para o desenvolvimento de habilidades e competências para buscar, compreender, avaliar e dar sentido a informações sobre saúde, visando o cuidado de si e de terceiros.

### O contraponto entre as concepções problematizadora e bancária da educação

Em sua obra seminal, *Pedagogia do Oprimido* ^26^, Paulo Freire cunha um termo bastante perspicaz para definir as práticas educativas centradas na mera transmissão de informações: “educação bancária”. Segundo o autor, a concepção bancária de educação está associada à condição passiva em que ambos os interlocutores (educador e educando) se encontram, em processos educativos reduzidos à mera transmissão de informações. O educador não questiona o conteúdo a ser transmitido, reduzindo o ato educativo ao “encher” o aluno de informações, tal qual um depósito bancário. O educando, por sua vez, aceita como verdade a informação transmitida, reproduzindo, de forma passiva, a condição estabelecida originalmente, de que estão inseridos em um mundo que é da forma como lhes é apresentado, e sobre o qual não há questionamentos ^26^.

É possível identificar, aqui, uma forte vinculação do modelo vertical-transmissionista da educação em saúde com a concepção bancária de educação, tal qual descrita por Paulo Freire. Vinculação que se estabelece, inicialmente, pelo fato de profissionais de saúde, em sua maioria, terem sua formação profissional construída em uma lógica “bancária” da educação e, ao concluírem seus cursos de graduação, geralmente se inserem num mercado de trabalho com pouco ou nenhum espaço para uma atuação crítica e problematizadora ^32^ ^,^ ^33^. E, por conseguinte, não encontram espaço ou estímulo para romperem com a lógica utilitarista de transmissão de conhecimento, reproduzindo-a junto aos usuários dos serviços, programas e sistemas de saúde ^34^.

Estabelece-se, portanto, um cenário de passividade, em que a educação bancária que serviu para a formação de base do educador (profissional de saúde) se reproduz em sua atuação profissional, limitando assim a possibilidade de o educando dar sentido, de forma crítica, ao conhecimento ali compartilhado (de maneira vertical e transmissionista) reduzindo, assim, a promoção da literacia em saúde ao acesso e à compreensão de informações ^1^.

Uma tendência recente, observada no conjunto das estratégias utilizadas para a promoção da literacia em saúde, é a produção de informações sobre saúde com “linguagem simples”. O conceito de linguagem simples surge, segundo Redish ^35^, na década de 1950, nos Estados Unidos, a partir de esforços para adequar textos de interesse público aos padrões de literacia fundamental da população estaduninense e que, a partir das décadas de 1970 e 1980, começaram a ser reproduzidos na Inglaterra e em outros países de língua inglesa. Inicialmente circunscritos ao conteúdo de textos legais (p.ex.: normas, regulamentos, termos e condições contratuais etc.), os esforços para a adoção de linguagem simples foram registrados, a partir da década de 1990, em diferentes países da Europa e, ao mesmo tempo, se tornaram mais abrangentes, ampliando seu escopo de atuação para setores como o comércio, a assistência social, a indústria e a saúde ^36^.

No setor de saúde, o movimento pela adoção de linguagem simples se aproxima, desde o final dos anos 1990, do campo em torno dos saberes e práticas associados à literacia em saúde, inicialmente apresentado como uma ação estratégica para a promoção da saúde e da autonomia junto aos usuários de serviços e programas de saúde ^37^ e, mais recentemente, visto com muito criticismo, em função das inúmeras experiências registradas na literatura crescente sobre o tema, nas quais se observa a não-ruptura com a lógica bancária da educação em saúde.

Segundo Dearfield et al. ^14^, as iniciativas para a promoção da literacia em saúde, em sua maioria, têm centrado esforços em instrumentalizar os profissionais de saúde com técnicas e ferramentas para a adoção de linguagem simples, com o intuito de garantir que as pessoas entendam o que eles recomendam e prescrevem durante as ações que coordenam. Para os autores, tal concepção reduz a educação e o desenvolvimento de habilidades a um modelo prescritivo-normativo, que enfatiza a importância da compreensão (mais que a significação) e adesão às instruções pelos usuários de serviços e programas de saúde, em vez de trabalhar habilidades e competências críticas que permitam que ambos os interlocutores (profissionais e usuários) atuem como decisores plenamente informados, engajados em um processo dialógico de significação comum ^14^.

Nesse ponto, novamente se observam as aproximações entre o pensamento freiriano e os fundamentos da perspectiva emancipatória da literacia em saúde, em que o educar não pode estar restrito à transferência de conhecimentos, com o intuito de aderência a instruções para mudanças de comportamento. Ensinar, dizia o educador, exige apreensão da realidade, respeito à autonomia do educando e à tomada consciente de decisões ^31^.

### Ensinar não é transferir conhecimento: exige apreensão da realidade, respeito à autonomia e à tomada consciente de decisões

Em *Pedagogia da Autonomia* ^31^, Paulo Freire apresenta uma reflexão aprofundada e, ao mesmo tempo, concisa, sobre os princípios que regem as práticas formativas promotoras de autonomia junto aos educandos. Partindo de uma distinção clara entre o formar e o treinar, o autor discorre sobre os limites da educação bancária - centrada na transferência de conhecimentos - para, em seguida, trazer ao debate os argumentos que justificam a ideia de que a leitura do mundo deve preceder a leitura da palavra. E, para tanto, enuncia uma série de “exigências” vinculadas à prática educativa problematizadora, as quais se aplicam igualmente à promoção da literacia em saúde em sua perspectiva emancipadora.

Uma primeira exigência é entender a apreensão da realidade como ato intrínseco ao educar, princípio que se vincula ao fato de não usarmos o conhecimento apenas para nos adaptarmos às condições impostas pela realidade, mas para mudá-la, transformá-la, recriando-a constantemente, condição que, segundo Freire, nos diferencia de outros animais e plantas ^31^.

Seguindo esse princípio, a adesão de um paciente a um tratamento ou estratégia para o cuidado de sua saúde, por exemplo, envolve mais elementos que o cumprimento à risca de uma prescrição: inclui a confiança no interlocutor (profissional de saúde ou outro ator), nas instituições pelas quais transitou para obter o atendimento em questão, sua capacidade de compreender as implicações de seguir ou não tais orientações, sua avaliação quanto à pertinência ou interesse nas mudanças de comportamento circunscritas às orientações e as escolhas que terá que fazer para adotá-las em seu cotidiano, entre outros fatores ^1^ ^,^ ^2^. Dentro desse quadro, o paciente deve ser capaz de argumentar sobre o propósito da prescrição, questioná-la e, mesmo, demandar que lhe sejam apresentadas alternativas que julgue serem mais adequadas às práticas de cuidado que pretende adotar, para si ou terceiros. É importante destacar, nesse sentido, que as orientações e as alternativas de cuidado à saúde apresentadas a esses pacientes pelos profissionais de saúde precisam considerar a (ou, preferencialmente, partir da) realidade sociocultural em que esses indivíduos se inserem, permitindo uma significação adequada ^1^.

A apreensão da realidade está, portanto, intimamente relacionada ao respeito à autonomia do educando por parte do educador e à possibilidade de que este último ofereça ao primeiro condições para a tomada consciente de decisões. A capacidade de intervir sobre a realidade ou modificá-la, e não apenas aceitá-la, por parte do educando (no caso acima, o usuário do serviço de saúde), está intimamente relacionada ao desenvolvimento de habilidades e competências específicas, objeto de estratégias de promoção da literacia em saúde, em sua perspectiva crítica e emancipatória. Essas estratégias, por sua vez, precisam estar intimamente relacionadas à realidade e ao contexto em que se inserem os educandos.

Um dos exemplos que mais bem evidenciam a necessidade de considerarmos os princípios freirianos na consolidação de uma perspectiva emancipadora para a promoção da literacia em saúde no Brasil é o fracasso das ações educativas sobre a dengue, nas diversas regiões do país, doença que continua afetando milhões de brasileiros, e com centenas de óbitos, ano após ano, há quase quatro décadas (considerando a primeira grande epidemia da doença, em 1986, na cidade do Rio de Janeiro). Segundo Rangel ^38^, parte da explicação reside no fato de as campanhas de comunicação e educação realizadas para o controle da dengue serem elaboradas em uma perspectiva centralizada, vertical e unidirecional, orientadas pelo princípio da transmissão de informações, podendo ou não considerar a adequação de linguagem ao grupo a que se destina. Para a autora, o objetivo principal dessas campanhas é a adesão do público às mensagens produzidas pelo emissor, orientando mudanças de hábito e comportamento para o enfrentamento da doença ^38^. No entanto, não contemplam estratégias que permitam às audiências a apreensão da realidade, principalmente no que diz respeito à responsabilidade dos atores e instituições envolvidos no controle da dengue/combate ao vetor, promovendo o pleno exercício da autonomia para uma tomada de decisão informada.

Em contraponto ao modelo transmissionista e verticalizado das campanhas sobre dengue realizadas no país, Paulo Freire questiona, em sua *Pedagogia da Autonomia* ^31^ (p. 17), o seguinte: “*por que não aproveitar a experiência que têm os alunos de viver em áreas da cidade descuidadas pelo poder público para discutir, por exemplo, a poluição dos riachos e dos córregos e os baixos níveis de bem-estar das populações, os lixões e os riscos que oferecem à saúde das gentes?*”. Reposicionando a pergunta do mestre para se pensar o enfrentamento da dengue, poderíamos dizer: por que não aproveitar a experiência que têm os alunos de viver em áreas da cidade descuidadas pelo poder público para discutir, por exemplo, estratégias de vigilância participativa, de base comunitária, de emergências de saúde pública como a dengue? Indagação que nos leva a considerar outro princípio freiriano, descrito em sua *Pedagogia do Oprimido* ^26^ (p. 71): “*ninguém liberta ninguém, ninguém se liberta sozinho: os homens se libertam em comunhão*”.

### “*Ninguém liberta ninguém, ninguém se liberta sozinho: os homens se libertam em comunhão*”

Um dos princípios mais conhecidos do pensamento freiriano, descrito em *Pedagogia do Oprimido* ^26^ e reproduzido no *caput* acima, vincula-se ao fato de não ser possível praticar a perspectiva emancipadora da educação - e, por que não dizer, da literacia em saúde - de maneira individualizada, dissociada do coletivo e do contexto social, ou de forma verticalizada, como nos “depósitos” de conhecimento próprios da concepção “bancária” da educação. Para que a educação seja, de fato, emancipadora, é preciso partir da apreensão da realidade e do reconhecimento das assimetrias historicamente impostas aos indivíduos e grupos da população. Somente assim será possível construir as alternativas necessárias para intervir e modificar a realidade que, no que tange ao conceito de literacia em saúde, está relacionada à melhoria das habilidades e competências que usamos para buscar, compreender, avaliar e dar sentido a informações para os cuidados de saúde de si e de terceiros.

Em *Pedagogia do Oprimido*, tal princípio é apresentado logo nas primeiras palavras do livro, onde o educador dedica sua obra “*aos esfarrapados do mundo e aos que neles se descobrem e, assim descobrindo-se, com eles sofrem, mas, sobretudo, com eles lutam*” ^26^ (p. 31). Descoberta que tanto pode se relacionar ao caráter humano de se colocar na condição do outro - o outrar-se - e descobrir um propósito para si próprio, ou ao reconhecimento de sua própria condição de esfarrapado. Em ambos os casos, a descoberta traz consigo sofrimento ao educador, mas, acima de tudo, o habilita e o instiga a lutar ao lado do educando, colocando-se como parte do problema e da solução - alusão que igualmente serve para se pensar o cuidado em saúde e o papel de profissionais e usuários dos serviços, programas e sistemas de saúde.

Libertar-se em comunhão significa descobrir a condição de esfarrapado de cada um e nela reconhecer a necessidade de criar os vínculos necessários para sua superação. O educador, nessa perspectiva, não deve responder pelo aprendizado do educando, da mesma forma que o profissional de saúde não deve responder pela saúde do paciente. Ambos os atores, ao reconhecerem as assimetrias impostas, os desafios a serem superados e seu papel na superação desse desequilíbrio, se engajam em um processo de promoção de autonomia e de aprendizado mútuo, libertando-se e educando-se (ou cuidando-se), assim, em comunhão.

Aqui, observa-se a estreita aproximação entre o pensamento freiriano e a perspectiva crítica da literacia em saúde, ou literacia em saúde crítica (*critical health literacy*). Segundo Sykes et al. ^39^, a perspectiva crítica da literacia em saúde ressignifica a concepção de empoderamento de Paulo Freire como um processo participativo de desenvolvimento comunitário, no qual os cidadãos tomam consciência das questões relacionadas ao cuidado (individual e coletivo) da saúde, participam ativamente nos espaços de diálogo crítico e se engajam na tomada de decisões sobre as estratégias de atenção, vigilância e promoção de saúde.

A concepção de uma perspectiva crítica da literacia em saúde, como contraponto ao conjunto de habilidades e competências circunscritas ao desenvolvimento da linguagem (domínio fundamental) ganha corpo a partir da obra seminal de Nutbeam ^5^, na qual o autor identifica três níveis de articulação, em torno dos quais essas habilidades e competências são demandadas: o nível funcional, em que o indivíduo lança mão de habilidades adquiridas ao longo de sua trajetória de vida, buscando o cuidado de sua saúde ou da de terceiros; o nível interativo (ou comunicativo), em que o indivíduo se vê inserido em uma comunidade, e nela constrói os vínculos necessários para o cuidado à saúde; e o crítico, em que o indivíduo se reconhece como cidadão e, ao mesmo tempo, membro de um grupo social, capaz de influenciar outros em sua tomada de decisão e de participar ativamente em processos e instâncias da sociedade a fim de promover a saúde, individual ou coletivamente.

Compreendida através dessa chave de análise, elaborada por Nutbeam ^5^, a perspectiva crítica da literacia em saúde representa um processo avançado de articulação que pode ser desenvolvido através de estratégias educativas emancipatórias, com enorme potencial de dotar os indivíduos de uma capacidade de significação crítica de informações e processos relevantes para o cuidado de sua saúde ^39^. Assim, vincula o pensamento freiriano - em particular sua pedagogia crítica-emancipatória - à promoção da literacia em saúde, em sua perspectiva mais crítica, junto a indivíduos e grupos no Brasil, na América Latina e no mundo.

## Considerações finais

A literacia em saúde, enquanto conjunto amplo de habilidades e competências relacionado às distintas formas de acessar, compreender, avaliar e dar sentido a informações sobre saúde, precisa ser considerada como elemento-chave para a promoção de autonomia e empoderamento de indivíduos e grupos da população, visando o cuidado de sua saúde e da de terceiros. Como qualquer habilidade ou competência, a literacia em saúde pode - e deve - ser desenvolvida a partir de iniciativas educativas, promovendo condições adequadas para o manejo, individual e coletivo, de situações de saúde, através da tomada de decisões informada e consciente.

Cabe destacar que o conceito de autonomia, apresentado e discutido em diferentes momentos ao longo do texto, precisa ser considerado dentro de uma perspectiva emancipadora da educação em saúde, e em hipótese alguma pode ser confundido com a transferência de responsabilidade sobre o cuidado a saúde para o indivíduo. Nessa concepção, um indivíduo com autonomia é aquele capaz de reconhecer, ao mesmo tempo, seu direito fundamental à saúde e seu papel enquanto cidadão, articulado a outros membros da comunidade e em diálogo permanente com as representações das estruturas sociais circunscritas a esse coletivo.

A partir do reconhecimento da centralidade da autonomia dos indivíduos nos processos de garantia do cuidado à saúde, individual ou coletivamente, as estratégias para a promoção da literacia em saúde precisam, também, estar alinhadas à garantia da participação da sociedade civil nos processos de formulação e gestão de serviços, programas e políticas de saúde.

Também é necessário considerar que as ações para o desenvolvimento da literacia em saúde de indivíduos e grupos da população precisam estar alinhadas a um processo de adaptação das instituições para operar em um contexto promotor das habilidades e competências associadas ao conceito de literacia em saúde. Essa perspectiva, que tem sido definida por muitos autores como literacia em saúde organizacional, está associada ao grau de organização institucional que permite que os indivíduos - trabalhadores e usuários - compartilhem informações e serviços de saúde em uma perspectiva equitativa e num quadro de significação comum, ajustado aos diferentes domínios da literacia em saúde.

No Brasil, pátria de Paulo Freire, cujo pensamento inspira e orienta as estratégias de promoção da literacia em saúde em sua perspectiva crítica, as estratégias para o desenvolvimento de habilidades e competências para buscar, compreender, avaliar e dar sentido a informações sobre saúde precisam contemplar, de maneira imediata e urgente, saberes e práticas sustentados pela pedagogia emancipatória freiriana e voltados para a promoção da literacia em saúde em sua perspectiva crítica. Sobretudo considerando o momento sociopolítico atual, marcado por polarizações extremadas e dificuldade de alcançar consensos mínimos para que avancemos em torno de um projeto de desenvolvimento social mais justo e equalitário, no qual os conceitos de empoderamento e ação social correm o risco de perder sua importância estratégica, é necessária e urgente a superação das falsas contradições entre as três acepções de *health literacy* adotadas no país (alfabetização, letramento e literacia em saúde), convergindo os incipientes esforços acadêmicos e dos atores da prática para a consolidação da perspectiva crítica e emancipatória da literacia em saúde no Brasil.
